# Alginate Oligosaccharides Protect Gastric Epithelial Cells against Oxidative Stress Damage through Induction of the Nrf2 Pathway

**DOI:** 10.3390/antiox13050618

**Published:** 2024-05-20

**Authors:** Samantha Acevedo, Alejandra A. Covarrubias, Paola Haeger, Floria Pancetti, Fadia Tala, Erwin de la Fuente-Ortega

**Affiliations:** 1 Laboratorio de Estrés Celular y Enfermedades Crónicas no Transmisibles, Universidad Católica del Norte, Coquimbo 1781421, Chile; samantha.acevedo@ce.ucn.cl; 2 Laboratorio de Neurotoxicología Ambiental, Departamento de Ciencias Biomédicas, Facultad de Medicina, Universidad Católica del Norte, Coquimbo 1781421, Chile; 3 Facultad de Ciencias Agropecuarias, Universidad del Alba, La Serena 1700000, Chile; 4 Laboratorio de Neurobiología de la Conducta, Departamento de Ciencias Biomédicas, Facultad de Medicina, Universidad Católica del Norte, Coquimbo 1781421, Chile; phaeger@ucn.cl; 5 Millennium Nucleus of Neuroepigenetics and Plasticity (EpiNeuro), Santiago 8370186, Chile; 6 Núcleo de Investigación en Prevención y Tratamiento de Enfermedades Crónicas no Transmisibles (NiPTEC), Universidad Católica del Norte, Coquimbo 1781421, Chile; ftala@ucn.cl; 7 Centro de Investigación y Desarrollo Tecnológico en Algas y Otros Recursos Biológicos (CIDTA), Facultad de Ciencias del Mar, Universidad Católica del Norte, Coquimbo 1781421, Chile; 8 Departamento de Biología Marina, Facultad de Ciencias del Mar, Universidad Católica del Norte, Coquimbo 1781421, Chile; 9 Instituto Milenio en Socio-Ecología Costera, SECOS, Santiago 7550000, Chile

**Keywords:** alginate oligosaccharides, AOS, Nrf2, Keap1, p62/SQSTM1, gastric diseases, GES-1

## Abstract

Gastric diseases represent a significant global public health challenge, characterized by molecular dysregulation in redox homeostasis and heightened oxidative stress. Although prior preclinical studies have demonstrated the cytoprotective antioxidant effects of alginate oligosaccharides (AOSs) through the Nrf2 pathway, whether such mechanisms apply to gastric diseases remains unclear. In this study, we used the GES-1 gastric cell line exposed to hydrogen peroxide (H_2_O_2_) as a damage model to investigate the impact of AOS on cell viability and its associated mechanisms. Our results revealed that pre-incubation with AOS for either 4 h or 24 h significantly improved the viability of GES-1 cells exposed to H_2_O_2_. In addition, AOS reduced the intracellular ROS levels, activating the Nrf2 signaling pathway, with increased Nrf2 protein and mRNA expression and a significant upregulation of the target genes HO-1 and NQO1. The activation of Nrf2 was correlated with decreased Keap1 protein expression and an increased level of the autophagy protein p62/SQSTM1, suggesting the activation of Nrf2 through a noncanonical pathway. This study suggests that AOS is a potential treatment for protecting gastric epithelial cells from oxidative stress by activating the p62/SQSTM1-Keap1-Nrf2 axis and laying the foundation for future investigations about its specific therapeutic mechanisms.

## 1. Introduction

Gastric diseases (GDs) significantly impact global human health [1]. They encompass a broad spectrum of conditions, including chronic gastritis, gastric ulceration, functional dyspepsia, and gastric cancer (adenocarcinoma), collectively resulting in 8 million deaths worldwide annually [2]. Both internal and external pathological factors can damage the gastric mucosa, promoting gastritis and ulceration, which may progress toward gastric cancer, which is the fifth most common cancer and the third cause of death from cancer in the world [2]. GDs compromise the function and structure of gastric epithelial cells, leading to increased permeability and facilitating the entry of the pathological bacteria *Helicobacter pylori* (*H. pylori*), which induces the progression of gastric cancer and gastric diseases [3,4,5,6]. Therefore, exploring new molecular targets and treatments to prevent cellular damage to the gastric epithelium is crucial in hindering the progression toward very complex diseases.

One of the primary pathological mechanisms of GDs that promote gastric epithelial cell injury is oxidative stress [7,8,9]. In a state of oxidative stress, cells lose their redox homeostasis, elevating levels of intracellular free radicals, such as reactive oxygen species (ROS), e.g., peroxide, superoxide (O_2_^·−^), or the hydroxyl radical (OH^·−^), thereby impacting the normal functions, growth, and viability of cells [10]. Several risk factors associated with GDs trigger oxidative stress, including nonsteroidal anti-inflammatory drugs, chronic alcohol consumption, cigarette smoking, and *H. pylori*, thus affecting the viability of gastric epithelial cells and altering their barrier and permeability functions [10,11,12]. Therefore, oxidative stress is a central molecular target concerning GDs, and using antioxidants as treatments (or adjuvants) may protect the gastric mucosa from oxidative damage [13,14]. The antioxidants derived from natural fruits and vegetables could be safer than supplements (vitamins) due to the potential harm caused by inappropriate antioxidant applications, which might scavenge the physiological role of ROS [15,16]. Recent preclinical studies have shown that alginate and alginate oligosaccharides (AOSs), naturally produced and extracted from brown seaweeds (Phaeophyceae), demonstrate beneficial health effects in other chronic pathologies [17,18,19,20]. AOSs may employ alternative antioxidant strategies to enhance the expression of endogenous antioxidant enzymes [21,22,23]. This suggests that AOSs have a potential use in therapies preventing oxidative stress in the gastric mucosa.

AOSs have demonstrated diverse biological activities in various preclinical models, including anti-inflammatory and antioxidative effects achieved through enhancing antioxidant enzymes [20,24]. For instance, in human umbilical vein endothelial cells, AOSs can enhance the activity of antioxidant enzymes, such as superoxide dismutase (SOD) and catalase (CAT) [21,22]. Recently, it was demonstrated that AOSs alleviate cisplatin-induced kidney oxidative stress by increasing the expression of antioxidant enzymes, including SOD and CAT [25], or exhibiting antitumor activity in osteosarcoma [26]. Additionally, Pan et al. (2021) reported that AOS can delay the aging process of the kidney by activating the nuclear factor erythrogen 2 associated factor 2 (Nrf2), a master regulator of the oxidative stress response that controls the expression of antioxidant enzymes, such as heme oxygenase-1 (HO-1) and NADPH quinone oxidoreductase 1 (NQO1), which prevent cells from oxidative damage [23]. Nrf2 is the primary regulator of the adaptive antioxidant response in cells and is widely expressed in metazoans [27,28,29,30], suggesting that a similar mechanism can act to protect gastric epithelial cells from oxidative stress.

This work used a normal (non-tumoral) human gastric epithelial cell line GES-1, which was subjected to damage induced by hydrogen peroxide (H_2_O_2_), as an in vitro model of gastric epithelial damage by oxidative stress. We aimed to assess the impact of AOSs on cell viability, oxidative stress levels, and the Nrf2 pathway. We found that the AOSs exhibited a protective effect on GES-1 exposed to oxidative stress, improving its viability and reducing its oxidative stress levels. Interestingly, the AOSs induced the Nrf2 pathway, in correlation with Keap1 degradation and an increased expression of p62/SQSTM1, suggesting the activation of Nfr2 by a noncanonical pathway. This study suggests that AOS could be a potential medical treatment to protect gastric epithelial tissue from oxidative stress by activating the p62/SQSTM1–Keap1–Nrf2 axis.

## 2. Materials and Methods

### 2.1. Materials

Alginate oligosaccharides (AOSs) (91.4%, ≤4000 Molecular Weight Da) were purchased from Qingdao BZ Oligo Biotech Co., Ltd. (Qingdao, China, Batch N° 2022032801AYF). Nrf2 inhibitor ML385 (#HY-100523) was purchased from MedChemExpress (Monmouth Junction, NJ, USA). Hydrogen peroxide was purchased from Merck Millipore (Darmstadt, Germany #107210). ML385 is soluble in DMSO, and hydrogen peroxide and AOS are soluble in aqueous solutions, such as water and culture media. MTS assay and Improm II^TM^ kit were purchased from Promega (Madison, WI, USA). Antibodies, anti-NRF2 (#D1Z9C), antiHO-1 (#E3F4S), and anti-KEAP1 (#D6B12) were purchased from Cell Signaling Technologies (Danvers, MA, USA). The antibodies anti-β-Actin (MA5-15739), anti p62/SQSTM1 (#PA5-20839), and anti-Nrf2 (#PA527882), as well as Alexa Fluor 488 anti-rabbit secondary antibody (#A11008), were obtained from Thermo Fisher Scientific (Eugene, OR, USA) The TRIZOL reagent (#15596018), DNase 1U (Turbo DNA-free^TM^ kit) (#AM1907), DCFH-DA (2′,7′-dichlorofluorescein diacetate) (#D6883), and Pierce^TM^ BCA Protein Assay kit (#23227) were acquired from Thermo Fisher Scientific.

### 2.2. Cell Culture and Oxidative-Stress-Induced Model

GES-1 cells were donated by Dr. Dawid Kidane-Mulat (Austin, TX, USA) and cultured according to previous publications [31]. Cells were maintained in high-glucose Dulbecco’s Modified Eagle Medium (DMEM) supplemented with 10% fetal bovine serum (FBS) (#SV30160.03, Hyclone-Cytiva, Pasching, Austria) and penicillin/streptomycin (1X) (#15140122, Thermo Fisher Scientific, Canada) at 37 °C in a humidified 5% CO_2_ incubator. For the oxidative-stress-induced model, the GES-1 cells were treated with different concentrations of hydrogen peroxide (50, 100, 200, and 400 μM) for 3 and 24 h, respectively, at 37 °C. We selected 100 μM H_2_O_2_ for 3 h for our subsequent experiments.

### 2.3. Cell Viability Assay

We used the tetrazolium reduction assay with MTS reagent to assess cell viability based on standard manufacturer protocols [32]. GES-1 cells (10,000 cells/mL) were seeded in 96-well plates for 24 h, and then the cells were treated with different concentrations of AOSs (100, 200, and 400 μg/mL) for 4 and 24 h. In another set of experiments, GES-1 cells were treated with H_2_O_2_ (50, 100, 200, and 400 μM) for 3 and 24 h, with six replicates being performed for each condition. After the elapsed time, 20 μL MTS/PMS was added to each well (final concentration of MTS was 333 μg/mL and that of PMS was 25 μM) for 1 h at 37 °C. The GES-1 cells that were pretreated with 400 μg/mL AOS for 4 and 24 h were also exposed to stimulation with H_2_O_2_ (50, 100, 200, and 400 μM) for 3 h. Additionally, to evaluate if the protective pathway is mediated by Nrf2 pathway, we used 5 μM of the Nrf2 inhibitor ML385 as pretreatment in combination with AOS and peroxide. After treatments, MTS/PMS was added, and absorbance was measured at 490 nm using a microplate reader (NOVOstar, BMG LabTech, Ortenberg, Germany). The viability was expressed as the percentage of reduced MTS; the absorbance of control cells represented 100% cell viability.

### 2.4. ROS Levels’ Quantitation

The intracellular ROS levels were measured with the fluorescent probe DCFH-DA (2′,7′-dichlorofluorescein diacetate) according to previous work [33]. Two plates were prepared to evaluate the ROS levels in cells that were preincubated with 400 μg/mL AOS for 4 and 24 h or were not preincubated. GES-1 cells (10,000 cells/mL) were seeded in 96-well plates (SPL, TCL group) and incubated for 24 h. A control with only medium and a control with 5 mM of antioxidant N-acetyl cysteine (NAc) were placed onto each plate. After the treatment, the cells were washed with PBS 1x, incubated with 10 μM DCFH-DA for 30 min at 37 °C, washed three times with PBS 1x, and exposed to different H_2_O_2_ concentrations (50, 100, and 200 μM) for 3 h. Finally, the fluorescence intensity of DCFH-DA was measured by NOVOstar (BMG LabTech, Ortenberg, Germany) with excitation and emission wavelengths of 485 nm and 535 nm, respectively. The fluorescence was expressed as a fold change, considering control with only medium as 1 unit.

### 2.5. Quantitative Reverse Transcription PCR (RT-qPCR)

We performed RT-qPCR according to previous work [34]. First, the GES-1 cells were incubated with media (control) or with different treatments, including only AOS (400 μg/mL for 18 h), only H_2_O_2_ (100 μM H_2_O_2_ for 3 h), or AOS with H_2_O_2_ (400 μg/mL AOS for 18 h, followed by 100 μM H_2_O_2_ for 3 h). After these incubations, the culture medium was discarded, and the cells were washed twice with PBS 1X and homogenized with TRIZOL to extract total RNA. Then, the total RNA was treated with DNase (1U) to eliminate contaminant DNA. The first-strand cDNA was synthesized with the Improm II^TM^ kit (Promega, Madison, WI, USA), the reaction tube contained 10 µg RNA, 3 mM MgCl_2_, 0.5 mM dNTPs, reaction buffer (50 mM Tris-HCl (with a pH of 8.3 at 25 °C), 75 mM KCl, and 10 mM DTT), 20 U (1 µL) of reverse transcriptase (Improm II^TM^, Promega, Madison, WI, USA), and nuclease-free water, reaching a final reaction volume of 5 µL. For the qPCR, specific primers for genes involved in the Nrf2 pathway and the housekeeping gene (GAPDH) with melting temperatures (Tm) of 60 °C and amplicons of approximately 100–200 bp are shown in Table 1. The qPCR reaction contained 5 µL of 2x SYBR green master mix (Kapa sybr^®^ fast, biosystems, Cedar Creek, TX, USA), complementary DNA (5 µL), 50 nM of each primer, and nuclease-free water until the final reaction volume reached 10 µL. Real-time PCR reactions were run with the Applied Biosystems StepOne™ system using the following amplification conditions: initial denaturation for 10 min at 95 °C, followed by 40 cycles of denaturation at 95 °C for 15 s, and annealing/extension at 60 °C for 30 s. Gene expression levels were normalized to the housekeeping gene GAPDH. To determine the relative mRNA levels between the expression of Nrf2 pathway genes (Nrf2, HO-1, NQO1) and the control group, the relative expression was quantified using the 2^−ΔCt^ method as previously calculated by [34] De la Fuente et al. (2019).

### 2.6. Cell Homogenization and Western Blot

We used the protocol of cell homogenization and Western blotting that has been described in previous works [34,36]. GES-1 cells (control or treated with AOS, H_2_O_2_, AOS/H_2_O_2_, or AOS/H_2_O_2_/ML385) were homogenized with 300 μL lysis buffer (20 mM MOPS/Tris at a pH of 7, 0.3 M sucrose, 2 mM EDTA, 2 mM EGTA, 1% NP-40, and 0.1% sodium dodecyl sulfate), and 3 μL protease inhibitors. The protein concentration was measured with a Pierce^TM^ BCA Protein Assay kit (Thermo Fisher Scientific), and 50 μg of protein was suspended in 3X loading buffer, denatured for 5 min at 90 °C, loaded in 10% sodium dodecyl sulfate–polyacrylamide gel electrophoresis (Minigel-BioRad), and transferred to 0.45 μm PVDF membrane (Thermo Fisher Scientific, #88518). The membranes were blocked with 5% nonfat milk in TBS-T (Tris-HCl 10 mM at a pH of 8, NaCl 150 mM, Tween 20 0.2%) for 1 h at room temperature and incubated for 18 h at 4 °C with the following primary antibodies: anti-β-actin (1:5000, Thermo Fisher Scientific); anti-p62 (1:1000, Thermo Fisher Scientific); anti-Nrf2 (1:1000, Cell Signaling Tech., Danvers, MA, USA); anti-HO-1 (1:1000, Cell Signaling Tech., Danvers, MA, USA); and anti-Keap1 (1:1000, Cell Signaling, Tech. Danvers, MA, USA). After being washed three times with TBS-T for 10 min, the blots were incubated with horseradish-peroxidase-conjugated secondary antibodies for 2 h at room temperature and washed three times for 15 min each with TBS-T. The blots were revealed via chemiluminescence using SuperSignal™ West Pico PLUS (Thermo Fisher Scientific), and images were captured using C-Digit and Odyssey M (LI-COR, Lincoln, NE, USA). The band intensity was analyzed with ImageJ version 1.53t.

### 2.7. Immunofluorescence and Quantitative Confocal Microscopy

GES-1 cells were seeded on coverslips in 24-well plates. The immunofluorescence analysis was performed using 70% confluent GES-1 cells. The cells (control or treated with AOS, H_2_O_2_, AOS/H_2_O_2_, or chloroquine control) were fixed with 4% paraformaldehyde for 10 min and washed with PBS1X. The cells were permeabilized with 0.2% Triton™ X-100 for 15 min and blocked with 2% BSA for 1 h at room temperature. Cells were incubated with primary antibodies anti p62/SQSTM1 (1:500, Thermo Fisher Scientific) and anti-Nrf2 (1:300, Thermo Fisher Scientific) overnight at 4 °C and washed three times for 15 min each time with PBS1X. This was followed by incubation with secondary antibody Alexa Fluor 488 (1:500, Thermo Fisher Scientific) for 1 h at room temperature and labeled with DAPI for 10 min. Cells were rinsed three times with PBS1X, and the coverslips were mounted on microscope slides using Fluoromount. The samples were examined with laser scanning confocal microscopy (LSCM-800) using 405 and 488 nm lasers and Plan-Apochromat 63×/1.46 oil immersion objective. The confocal images were obtained with a size of 1024 × 1024 pixels (202.83 μm^2^) and processed to produce regions of interest (ROIs) per cell using the software ZEN-2.1. After eliminating the background using a threshold for Nrf2 and p62 image, their expression was calculated as the integrated mean intensity of green fluorescence (488 channel) per cell (*n* = 109–177 for Nrf2, and *n* = 72–115 for p62). For each treatment, the average of integrated intensity was calculated using the following formula:

Average Integrated Intensity=(ΣNPc×Mean intensity)/n

where NPc = number of Pixels per cell, MIc = mean intensity per cell, and *n* = number of cells.

Additionally, we evaluated ROS levels by in vivo confocal microscopy assay. GES-1 cells (100,000 cells/mL) were seeded in a glass bottom cell culture dish (NEST, Labmed, Wuxi, China) and incubated for 24 h. The GES-1 cells were incubated with a normal medium (control), and different treatments (100 μM H_2_O_2_, 400 μg/mL AOS + 100 μM H_2_O_2_, or 400 μg/mL AOS + 100 μM H_2_O_2_ + 5 μM ML385). After that, the cells were loaded with 10 μM DCFH-DA for 30 min at 37 °C, washed three times with PBS1X, and maintained in HBSS 1X recoding buffer. The samples were examined with LSCM-800 using excitation wavelengths of 485 nm (emission of 536 nm) and a Plan-Apochromat 63×/1.46 oil immersion objective. After background elimination using a threshold for 488 channel, the average integrated intensity was calculated for 80 to 142 cells per treatment and expressed as fold change relative to the control.

### 2.8. Statistical Analysis

All experiments were performed in triplicate, and results were estimated as mean ± S.E.M. Statistical parametric analyses were performed with Graph Pad Prism 8. Data were compared using a *T*-test for the comparison of two groups and one-way ANOVA for the comparison between groups, with a significance level of *p* (* *p* < 0.05, ** *p* < 0.05, *** *p* < 0.03, **** *p* < 0.0001). Additionally, the software used the “Identify Outliers” analysis to detect outliers in the experiments.

## 3. Results

### 3.1. AOS Improved Viability of Gastric Epithelial Cell Line GES-1 Exposed to Hydrogen Peroxide

**Figure 1:**
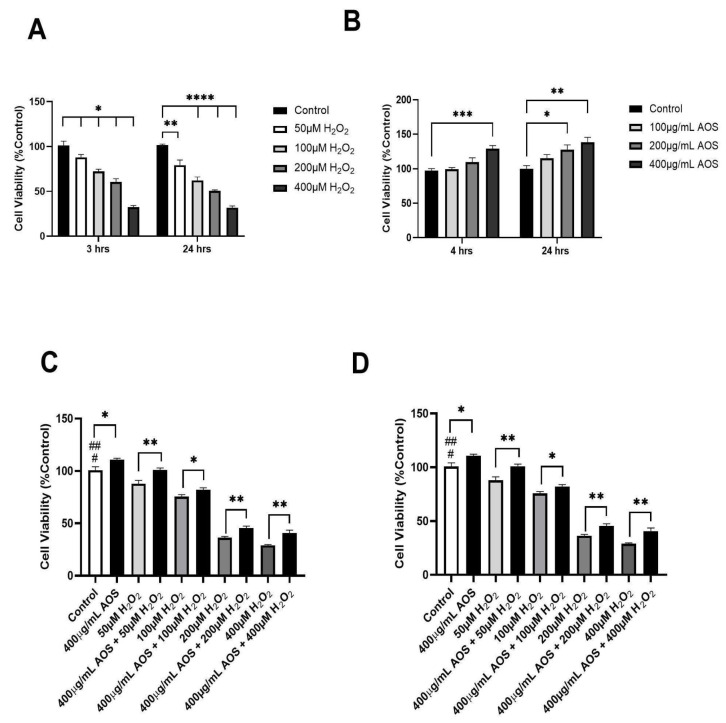
**AOS protects the GES-1 cell line exposed to H_2_O_2_.** (**A**) Cell viability of GES-1 exposed to different concentrations of H_2_O_2_ (50, 100, 200, and 400 μM) for 3 and 24 h. (**B**) Cell viability of GES-1 incubated with different concentrations of AOS (100, 200, and 400 μg/mL) for 4 and 24 h. (**C**) Cell viability of GES-1 preincubated with 400 μg/mL AOS for 4 h and exposed to different concentrations of H_2_O_2_ (50 to 400 μM) for 3 h. (**D**) Cell viability of GES-1 preincubated with 400 μg/mL AOS for 24 h and exposed to different concentrations of H_2_O_2_ (50 to 400 μM) for 3 h. Cell viability was expressed as a percentage of cell viability relative to control. Data are presented as the means ± S.E.M. for *n* = 3 independent experiments. ^#^ *p* < 0.05 H_2_O_2_ (50 and 100 μM) vs. Control, ^##^ *p* < 0.0001 H_2_O_2_ (200 and 400 μM) vs. Control, * *p* < 0.05 AOS vs. Control, * *p* < 0.05 H_2_O_2_ vs. AOS + H_2_O_2_, ** *p* <0.01 H_2_O_2_ vs. AOS + H_2_O_2_, and *** *p* < 0.003 vs. Control, **** *p* < 0.0001 H_2_O_2_ (100, 200 and 400 μM) vs. Control.

Previous research has demonstrated that AOSs protect against oxidative stress in various cell lines [22,25,37,38]. Based on these previous research studies, we set the exposition of H_2_O_2_ (3 or 24 h) and the pre-incubation times for the AOSs (4 and 24 h) and evaluated their effects on the viability of the human gastric epithelium cell line GES-1 (Figure 1A–D). As we expected, as the H_2_O_2_ concentration increased, the viability of GES-1 decreased significantly at 3 and 24 h of incubation with H_2_O_2_, with IC50 values of 300 μM and 200 μM, respectively (Figure 1A). In contrast, the AOSs did not reduce the viability of GES-1 but improved its viability at 4 and 24 h of incubation compared with that of the control (Figure 1B). Furthermore, the preincubation of GES-1 with 400 μg/mL AOS for 4 and 24 h significantly (*p* < 0.05) protected the cells against exposition to H_2_O_2_ (Figure 1C,D). When the GES-1 cells were preincubated with the AOSs for 4 h, their viability improved by 7–17% (*p* < 0.05 and *p* < 0.01), even when exposed to 50 to 400 μM peroxide, compared to that of the control without AOSs (Figure 1C). Similar results were observed after preincubation with the AOSs for 24 h, which significantly increased the cell viability of GES-1 (7% to 14%, *p* < 0.05 and *p* < 0.01) in the presence of 50 to 400 μM peroxide compared to that of the control cells without AOS exposure (Figure 1D). Therefore, preincubation with AOS for 4 or 24 h can improve the viability of GES-1 exposed to peroxide.

### 3.2. AOS Decreased the Intracellular ROS Levels of GES-1 Cells Exposed to Hydrogen Peroxide

**Figure 2:**
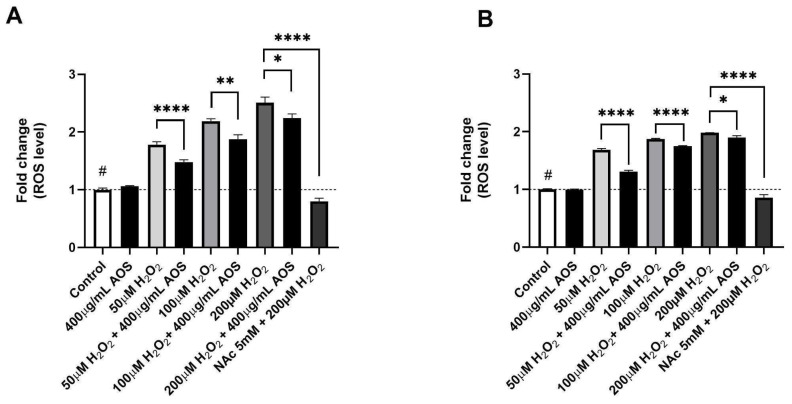
**AOS decreased intracellular ROS levels of GES-1 exposed to hydrogen peroxide**. (**A**) Fold change in ROS levels in GES-1 cells pre-treated with AOS (400 μg/mL) for 4 h and exposed to different H_2_O_2_ concentrations (50, 100, and 200 μM) for 3 h. (**B**) Similar experiment to (**A**), but the AOSs were preincubated for 24 h and, after that, incubated with H_2_O_2_ concentrations (50, 100, and 200 μM) for 3 h. In both experiments, 5 mM N-acetylcysteine (NAc) was used as a positive control. The fluorescence of the DCFH-DA reagent was expressed as the fold change in fluorescence intensity concerning the basal condition (control). Data are expressed as the means ± S.E.M for *n* = 3 independent experiments. ^#^ *p* < 0.0001 H_2_O_2_ vs. Control, * *p* < 0.05 H_2_O_2_ vs. AOS + H_2_O_2_, ** *p* < 0.01 H_2_O_2_ vs. AOS + H_2_O_2_, **** *p* < 0.0001 H_2_O_2_ vs. AOS + H_2_O_2_, and **** *p* < 0.0001 H_2_O_2_ vs. H_2_O_2_ + NAc.

Previous reports have demonstrated that AOS protects cells through their antioxidant activity [22,37,39]. To evaluate whether AOSs have antioxidant activity in GES-1, we quantified the intracellular ROS levels using a fluorescent probe (DCFH-DA) in GES-1 preincubated with AOSs that was exposed or was not exposed to H_2_O_2_ (Figure 2A,B). The results showed that GES-1 preincubated with 400 μg/mL AOS for 4 h significantly reduced (*p* < 0.0001–*p* < 0.05) its intracellular ROS levels after exposure to peroxide compared to GES-1 that was not treated with AOS (Figure 2A). A similar significant effect (*p* < 0.0001–*p* < 0.05) was observed in GES-1 preincubated with 400 μg/mL AOS for 24 h, in which the intracellular ROS level of GES-1 decreased compared to that of the cells that were not treated with AOS (Figure 2B). As a positive control, we used N-acetylcysteine (NAc), which is a well-known pharmacological and antioxidant mechanism [40,41]. Five mM NAc was able to significatively recuperate (*p* < 0.0001) the normal intracellular ROS levels in cells exposed to 200 μM of peroxide. Thus, GES-1 preincubated with the AOS (4 or 24 h) decreased its intracellular ROS levels when exposed to peroxide.

### 3.3. AOS Protected GES-1 Cells Exposed to Hydrogen Peroxide through the Nrf2 Pathway

**Figure 3:**
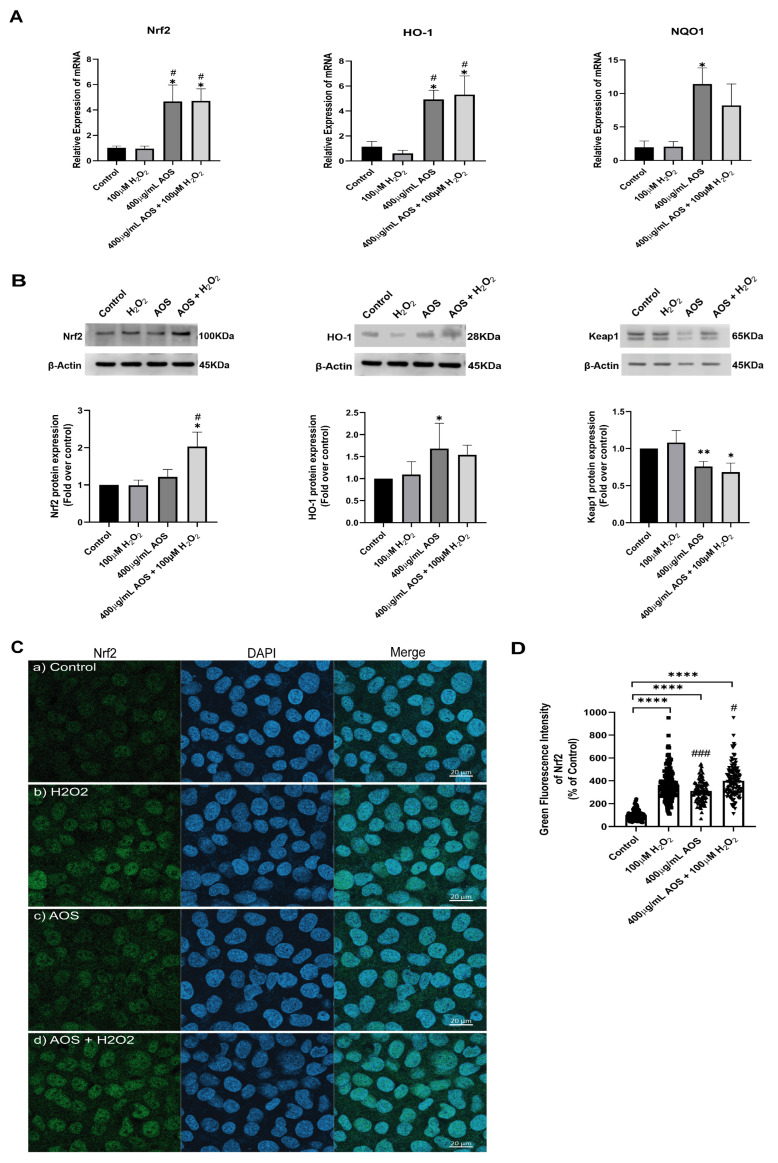
**AOS induce the Nrf2 pathway in the GES-1 cell line exposed to H_2_O_2_.** (**A**) Relative expression (fold change of 2^−ΔCt^) of Nrf2, HO-1, and NQO1 by RT-qPCR in GES-1 cells. The cells were exposed to medium, H_2_O_2_, AOS, and AOS plus H_2_O_2_ at the concentration indicated in the figure, and the mRNAs of these specific genes were analyzed by RT-PCR. *n* = 3 independent experiments. (**B**) Relative expression of Nrf2, HO-1, and Keap1 proteins by Western blot in GES-1. The cells were exposed to medium, H_2_O_2_, AOS, and AOS plus H_2_O_2_ at the same concentration indicated in (**A**), and the protein expression of Nrf2, HO-1, and Keap1 was normalized with β-actin and showed a fold change over control. *n* = 4 independent experiments. (**C**) Expression of Nrf2 by immunofluorescence. GES-1 cells were incubated with (a) medium, (b) 100 µM H_2_O_2_, (c) 400 ug/mL AOS, and (d) 400 µg/mL AOS and 100 µM H_2_O_2_; after incubation, the cells were fixed and immunolabeled with anti-Nrf2 (and Alexa Fluor 488, Green) and DAPI (Blue), and a representative image was acquired by confocal microscopy. Scale bar: 20 µm. (**D**) Nrf2 level expression by quantitative fluorescence. The Nrf2 level expression was quantified as the average integrated intensity calculated for each condition of (C). *n* = 109 to 177 cells per treatment. Data are expressed as the means ± S.E.M. * *p* < 0.05 vs. Control, ** *p* < 0.01 vs. Control, **** *p* < 0.0001 vs. Control, ^#^ *p* < 0.05 H_2_O_2_ vs. AOS and AOS + H_2_O_2_, ^###^ *p* < 0.003 H_2_O_2_ vs. AOS.

**Figure 4:**
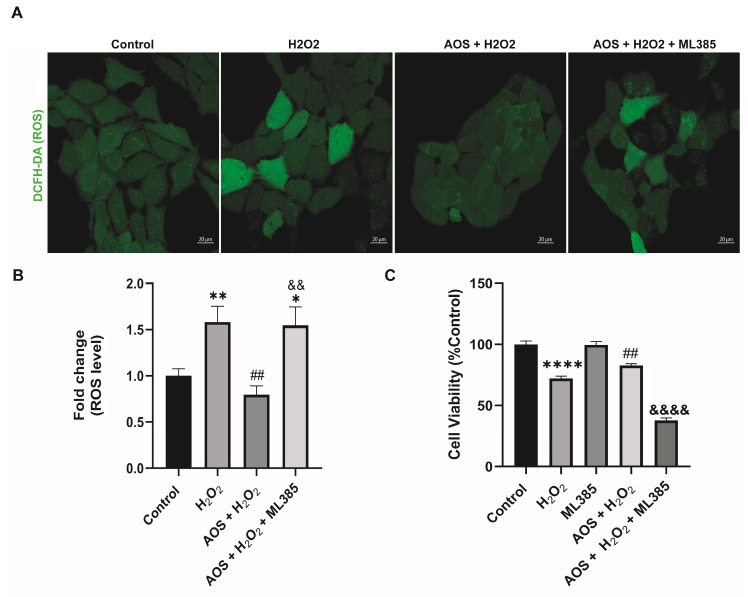
**Specific Nrf2 inhibitor (ML385) affects the protective effect of AOS in GES-1 cells exposed to H_2_O_2_.** (**A**) ML385 increases the oxidative stress of GESl-1 cells exposed to AOS and peroxide. GES-1 cells were treated with various conditions: only medium (control), 100 mM H_2_O_2_ for 3 h (H_2_O_2_), 100 mM H_2_O_2_ 3 h after preincubation with 400 μg/mL AOS for 24 h (AOS + H_2_O_2_), 100 mM H_2_O_2_ 3 h after preincubation with 400 μg/mL AOS plus 5 μM ML385 for 24 h (AOS + H_2_O_2_ + ML385). The ROS levels were visualized using the DCFH-DA fluorescent probe, and images were acquired by confocal microscopy. (**B**) The fluorescence of the DCFH-DA reagent was quantified as the average integrated intensity calculated for each condition shown in (**A**). The fluorescence intensity was expressed as the fold change in fluorescence relative to the control. Data are expressed as the means ± S.E.M with *n* = 80 to 142 cells per treatment. * *p* < 0.05 Control vs. AOS + H_2_O_2_ + ML385. ** *p* < 0.01 H_2_O_2_ vs. Control. ^##^ *p* < 0.01 H_2_O_2_ vs. AOS + H_2_O_2_. ^&&^ *p* < 0.01 AOS + H_2_O_2_ vs. AOS + H_2_O_2_ + ML385. (**C**) Effects of ML385 on cell viability of GES-1. GES-1 cells were preincubated with medium (control), 100 mM H_2_O_2_ for 3 h (H_2_O_2_), 5 μM ML385 for 24 h (ML385), 100 mM H_2_O_2_ 3 h after preincubation with 400 μg/mL AOS for 24 h (AOS + H_2_O_2_), 100 mM H_2_O_2_ 3 h after preincubation with 400 μg/mL AOS plus 5 μM ML385 for 24 h (AOS + H_2_O_2_ + ML385). Cell viability was expressed as a percentage of cell viability relative to control. Data are presented as the means ± S.E.M. for *n* = 3 independent experiments. **** *p* < 0.0001 Control vs. H_2_O_2_. ^##^ *p* < 0.01 H_2_O_2_ vs. AOS + H_2_O_2_. ^&&&&^ *p* < 0.0001 AOS + H_2_O_2_ vs. AOS + H_2_O_2_ + ML385.

Previous reports have shown that AOS protects against oxidative stress by activating the Nrf2 pathway [22,37], suggesting a similar mechanism may be triggered in GES-1. Under basal conditions, Nrf2 is permanently degraded by binding to its inhibitor Keap1 (Kelch-like ECH-associated protein 1), but in the presence of an oxidative stress stimulus, Nrf2 is stabilized, its expression increases, and it is translocated to the nucleus to turn on target genes, including HO-1 and NQO1 [27]. To access this mechanism, we used an RT-qPCR to measure the expression of the transcription factor Nrf2 and its target genes HO-1 and NQO1 (Figure 3A). We found that AOS alone or together with H_2_O_2_ induced a significant level (*p* < 0.05) of mRNA expression for Nrf2, HO-1, and NQO1 in GES-1 cells compared to that of the control (Figure 3A). The Nrf2 increased 4.6-fold compared to the control (*p* = 0.0186 AOS + H_2_O_2_ vs. control), and the target genes HO-1 and NQO1 increased 4.7- and 11.3-fold compared to the control, respectively (*p* = 0.0113 and *p* = 0.0216 AOS vs. control). Consistent with this result, AOS alone or in the presence of peroxide was able to significantly induce (*p* < 0.05) the expression of Nrf2 and the OH-1 protein, which were measured by Western blotting (Figure 3B). Furthermore, AOS alone or in the presence of H_2_O_2_ reduced the expression of Keap1 protein (Figure 3B). Additionally, the same conditions generated a significant increase in the translocation of Nrf2 in the nucleus of GES-1 cells (210.1% ± 8.9, *p* < 0.0001 and 299.4% ± 12.5, *p* < 0.0001), which was detected by immunofluorescence and image quantification (Figure 3C,D). In GES1, H_2_O_2_ at concentrations of 100 mM and 400 mM induced Nrf2 nuclear translocation within 3 h, but not within 6 h, which correlates with the onset of cellular damage (pycnotic nucleus) (Supplementary Figure S1). Furthermore, to assess whether AOS protect GES-1 cells from H_2_O_2_ via the Nrf2 pathway, we administered a specific inhibitor of Nrf2 called ML385 (Figure 4). We observed that 5 mM ML385 significantly increased the oxidative stress of GES-1 exposed to both AOS and peroxide, while the AOS treatment decreased the ROS levels of GES-1 exposed to peroxide (Figure 4A,B). Additionally, we found that ML385 significantly affected (*p* < 0.0001) the cell viability of GES-1 exposed to AOS and H_2_O_2,_ compared to the group treated with AOS plus H_2_O_2_ (Figure 4B). Furthermore, these effects corresponded with a qualitative decrease in Nrf2 and HO-1 protein levels in GES-1 treated with ML385 (Supplementary Figure S2). Therefore, these results indicate that AOSs protect cell viability and decrease oxidative stress in GES-1 cells exposed to peroxide, specifically through the Nrf2 pathway.

### 3.4. AOS Induced the Expression of p62/SQSTM1 in GES-1 Cells Exposed to Peroxide

**Figure 5:**
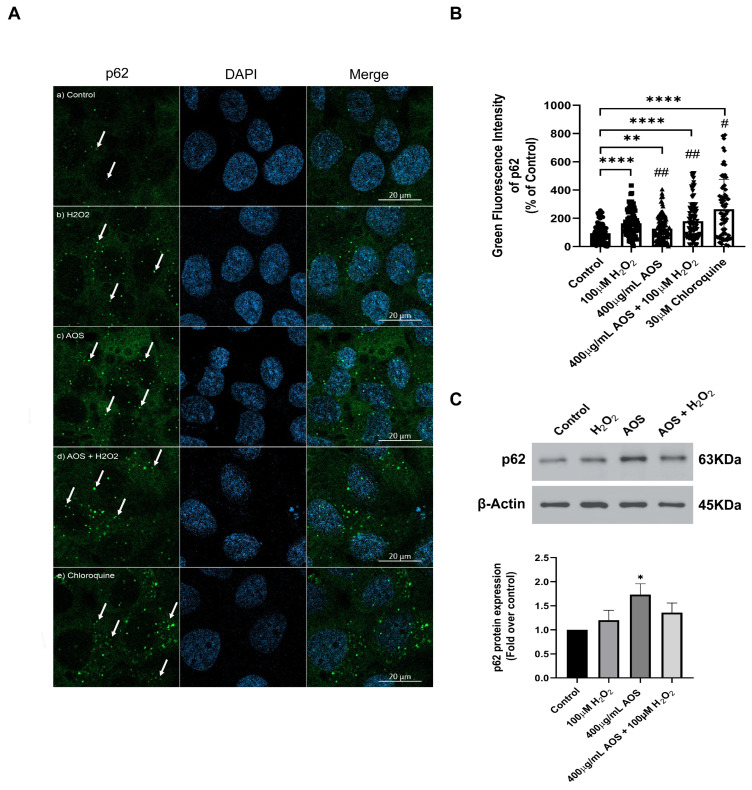
**Effect of AOS on the p62/SQSTM1 protein expression in GES-1 cell line exposed to H_2_O_2_.** (**A**) AOS-induced p62/SQSTM1 expression in GES-1. GES-1 cells were pretreated for 24 h with medium (a) or 400 µg/mL AOS (c,d) and exposed for 3 h to 100 µM peroxide (b,d) or for 5 h to 30 µM chloroquine (e). The cells were fixed and immunolabeled with anti-p62 (and secondary Ab Alexa Fluor 488, Green) and DAPI (Blue). Images were acquired with confocal microscopy. Scale Bar: 20 µm. (**B**) Expression of p62/SQSTM1 calculated by the integrated intensity of green fluorescence. The fluorescence intensity was expressed as a percentage relative to the control with *n* = 72 to 115 cells per treatment. (**C**) Western blotting shows the expression levels of p62 after normalization with β-actin for *n* = 4 independent experiments. Data are expressed as the means ± S.E.M. * *p* < 0.05 vs. Control, ** *p* < 0.0049 Control vs. AOS, **** *p* < 0.0001 Control vs. H_2_O_2_ **** *p* < 0.0001 Control vs. AOS + H_2_O_2_, **** *p* < 0.0001 Control vs. Chloroquine. ^#^ *p* < 0.0001 H_2_O_2_ vs. Chloroquine, ^##^ *p* < 0.0001 Chloroquine vs. AOS and AOS + H_2_O_2_.

The noncanonical mechanism that activates Nrf2 mediates the degradation of Keap1 by p62/SQSTM1, a multifunctional stress-inducible scaffold protein that links the Nrf2 pathway and autophagy [42,43,44]. p62/SQSTM1 can interact with Keap1, promoting its autophagic degradation [44,45]. To assess whether AOS can induce the expression of p62/SQSTM1 in GES-1, we measured the protein level expression of the p62/SQSTM1 protein by immunofluorescence and confocal imaging (Figure 5A). The images showed that H_2_O_2,_ AOS, and AOS plus H_2_O_2_ increased the fluorescence intensity and the number of green dots of p62/SQSTM1 in GES-1 compared to the control (only the medium) (see the arrows in Figure 5A). Also, we quantified the expression of p62/SQSTM1, calculating the average of the integrated intensity of p62/SQSTM1 fluorescence (Figure 5B). We found that 400 µg/mL of AOS alone or in the presence of H_2_O_2_ significantly enhanced (32.4% ± 11.4%, *p* = 0.0049, and 84.9% ± 15.2, *p* < 0.001, respectively) the expression of p62/SQSTM1 (green dots) compared to the control cells with only the medium (Figure 5B). As a positive control, we treated the GES-1 with 30 mM chloroquine, an autophagy inhibitor, increasing the labeling and integrated intensity of p62/SQSTM1 (Figure 5A,B). In addition, we analyzed the effect of AOS on the p62/SQSTM1 protein expression by Western blotting (Figure 5C). The result showed that the treatment with AOS (400 µg/mL) significantly (*p* = 0.0176) increased p62/SQSTM1 protein expression levels, while the treatment with H_2_O_2_ and AOS plus H_2_O_2_ showed a tendency to increase their expression. Therefore, these results showed that AOS increased the expression of the p62/SQSTM1 protein, suggesting the involvement of this pathway in the activation of NfR2.

## 4. Discussion

Oxidative stress is highlighted as a primary pathological mechanism in gastric diseases (GDs), impacting the function and integrity of the gastric epithelial cells within the gastric mucosa. This study explored the effects of alginate oligosaccharides (AOSs) on the human gastric epithelial cell line GES-1 exposed to peroxide (H_2_O_2_). Our findings revealed that AOS effectively protected against oxidative stress, enhancing the viability of GES-1 by mitigating ROS levels. This protective effect of AOS was mediated by a mechanism involving the Nrf2 signaling pathway, in which Nrf2 was activated by a noncanonical pathway involving p62/SQSTM1. These results highlight the potential utility of AOS as an antioxidant to prevent cellular damage induced by oxidative stress in gastric diseases.

Our results highlight, for the first time, that AOS can improve the viability of the gastric epithelial cell line GES-1 when exposed to peroxide. We used commercially available AOS (Qingdao BZ Oligo Biotech Co.), which has been used in several previous experiments [22,39,46]. Consistent with previous findings, we observed a significant improvement in the cellular viability of GES-1 cells when preincubated with AOS for 4 or 24 h before being exposed to peroxide. Moreover, AOS preincubation led to a significant reduction in intracellular ROS levels in GES-1 cells exposed to peroxide compared to those not preincubated with AOS. This suggests that AOS enhances gastric epithelial cells through an antioxidant mechanism. A 4 h preincubation with AOS was sufficient to confer protection against peroxide to GES-1 cells, comparable to the protection provided by 24 h of preincubation. These results are aligned with those of previous studies demonstrating the antioxidant bioactivity of AOS in various cell types, including umbilical vein endothelial cells in humans (HUVEC), neuron-like PC12 cells, and the kidney tissue of mice [22,25,37]. Indeed, Zhao et al. (2020) showed that overnight preincubation with AOS (50–800 mg/mL) improved cell viability and reduced intracellular ROS levels in human umbilical vein endothelial cells exposed to 400 μM peroxide [22]. Similarly, Tusi et al. (2011) demonstrated that preincubation with AOS (500–800 mg/mL) for 24 h improved the cell viability of rat-derived PC12 cell lines exposed to 150 μM H_2_O_2_ [37]. In a mouse model, AOS (5 mg/mL) administered via gavage for 3 weeks improved kidney and liver function in mice damaged by chemotherapy drugs, such as cisplatin, compared to that of controls [25]. Additionally, AOS administered at doses of 50 to 150 mg/Kg/d for four weeks alleviated D-galactose-induced cardiac aging [39]. Therefore, our results, along with those of other studies, indicate that AOS exhibits cellular antioxidant activity in human GES-1 cells and other cell lines, as well as in different animal models, suggesting AOS has a conserved antioxidant mechanism.

Previous studies have shown that AOS can induce the Nrf2 signaling pathway, which plays a critical role as a regulator of the cell defense mechanism against oxidative stress by controlling the expression of several cellular protective proteins [23,25,37]. Nrf2 is a transcription factor that, under basal conditions, is negatively regulated by Keap1, an adaptor protein for the Cul3 E3 ubiquitin ligase, which is responsible for the ubiquitination and proteasomal degradation of Nrf2 [27,47]. However, many inducers can promote Nrf2 protein levels and activate the pathway [48,49]. Upon activation, Nrf2 is released from Keap1, resulting in the stabilization and activation of Nrf2, which translocates to the nucleus, triggering the transcription of cytoprotective genes that contain an antioxidant response element (ARE), including OH-1, NQO-1, and the Nfr2 gene itself [27,50]. In agreement with this, we found the first piece of evidence that the preincubation of 400 μg/mL AOS for 24 h alone and that of AOS co-incubated with peroxide were able to induce the expression of Nrf2 mRNA and Nrf2 protein, accompanied by an increased expression of the Nrf2 target genes OH-1 and NQO1 in GES-1, compared to incubation with only the medium or only the peroxide. The incubation with H_2_O_2_ for 3 h could only activate Nrf2 translocation. However, at this time, H_2_O_2_ did not induce the transcription of Nrf2, OH-1, and NQO-1 mRNAs or the translation of Nrf2 and OH-1 proteins. This is likely because eukaryotic cells’ gene expression (transcription and translation) requires longer than the posttranslational process (Nrf2 translocation) [51]. Furthermore, to assess whether the effects of AOSs were specifically mediated by Nrf2, we employed a specific Nrf2 inhibitor known as ML385 [52]. This inhibitor affects the transcriptional activity of Nrf2 and has been utilized to elucidate the involvement of the Nrf2 pathway in the mechanism of action of various substances [53,54,55,56,57]. Our findings indicate that ML385 affected the cell viability of GES-1 treated with AOS and exposed to peroxide, increasing intracellular ROS levels and affecting the expression protein levels of Nrf2 and HO-1. These results demonstrate that AOS can protect the GES-1 from oxidative stress involving the Nrf2 pathway.

In addition, we further investigated the potential activation of Nrf2 at the level of the Keap1 protein (a redox-sensitive E3 ubiquitin ligase), which can be activated by conformational changes or by degradation through a noncanonical pathway of Nrf2 activation [48,58]. We found that AOS alone or AOS in the presence of peroxide reduced Keap1 protein levels, suggesting that AOS can stimulate the noncanonical activation of Nrf2. The noncanonical mechanism of Nrf2 activation is mediated by p62/SQSTM1 (Sequestosome 1) and autophagy [45,48]. In this pathway, p62/SQSTM1 interacts with Keap1, inducing its degradation by autophagy, which produces the stabilization of Nrf2, which activates ARE-bearing genes, including the p62/SQSTM1 gene itself [48,59]. Consistent with this, we found that AOS alone or in the presence of peroxide induced a significant expression of p62/SQSTM1 by immunofluorescence and Western blotting compared to a control, thus suggesting that AOS can induce this noncanonical pathway to activate Nrf2. There is recent evidence that several natural extracts from different sources, including ginseng and *Vulpia ciliata*, and purified substances, such as the polyphenol resveratrol and the sulfated polysaccharide fucoidans, can induce the noncanonical p62/SQSTM1 pathway to protect different cell lines [60,61,62,63]. It is still unclear how different substances, such as resveratrol and polysaccharides, fucoidans, and AOS (which is covered in this paper), can activate p62/SQSTM1. The p62/Keap1/Nrf2 axis is conserved and involves a complex pathway in metazoans [29,30]. It is possible that these diverse substances can trigger the axis by different mechanisms that need to be further investigated. Future research will focus on how AOS from the extracellular space can induce this axis in GES-1. These current findings showed that AOS can trigger the p62/Keap1/Nrf2 axis in gastric epithelial cell lines, representing a potential new therapeutic target to combat oxidative stress in gastric diseases.

In summary, our study reveals, for the first time, that AOS can protect the gastric epithelial cell line (GES-1) from peroxide-induced oxidative stress. Given that oxidative stress is a central pathological mechanism in gastric diseases (GDs), our findings suggest that AOS shows promise as a potential treatment or adjuvant in addressing these significant pathologies. A functional food formulation with AOS could potentially protect the gastric mucosa epithelium from oxidative damage. While several medicinal plants present direct antioxidant activity with fewer adverse effects and could serve as alternative treatments for ulcer diseases [64], AOS can operate through a distinct mechanism, as shown here, involving the p62/SQSTM1/Keap1/Nrf2 axis to increase antioxidant enzymes and prevent damage to the gastric epithelium cell line. Future studies using animal models of GDs are required to establish these pathologies’ safety and potential protective effects.

## Figures and Tables

**Table 1:** Nucleotide sequences of the forward (F) and reverse (R) primers used for qRT-PCR of genes involved in the Nrf2 pathway and the housekeeping gene GAPDH. The sequences of primer pairs used were reported by [35].

Gene/Accession Number	Forward	Reverse
HO-1/NM_002133.3	5′-GAG TGT AAG GAC CCA TCG GA-3′	5′-GCC AGC AAC AAA GTG CAA G-3′
NQO-1/NM_000903.3	5′-TCC TTT CTT CTT CAA AGC CG-3′	5′-GGA CTG CAC CAG AGC CAT-3′
NRF2/NM_006164.5	5′-TCT TGC CTC CAA AGT ATG TCA A-3′	5′-ACA CGG TCC ACA GCT CAT C-3′
GAPDH/NM_002046.7	5′-AAG GTG AAG GTC GGA GTC AA-3′	5′-AAT GAA GGG GTC ATT GAT GG-3′

## Data Availability

Data are contained within the article and Supplementary Materials.

## Supplementary Materials

The following supporting information can be downloaded at https://www.mdpi.com/article/10.3390/antiox13050618/s1.
