# Supplementary material for: Alginate Oligosaccharides Protect Gastric Epithelial Cells against Oxidative Stress Damage through Induction of the Nrf2 Pathway
Source: Antioxidants (Basel). 2024 May 20;13(5):618. doi: 10.3390/antiox13050618 (PMC11117588; doi:10.3390/antiox13050618)
Supplement: Supplementary file 1 [file antioxidants-13-00618-s001.zip › antioxidants-2879022-supplementary.pdf]

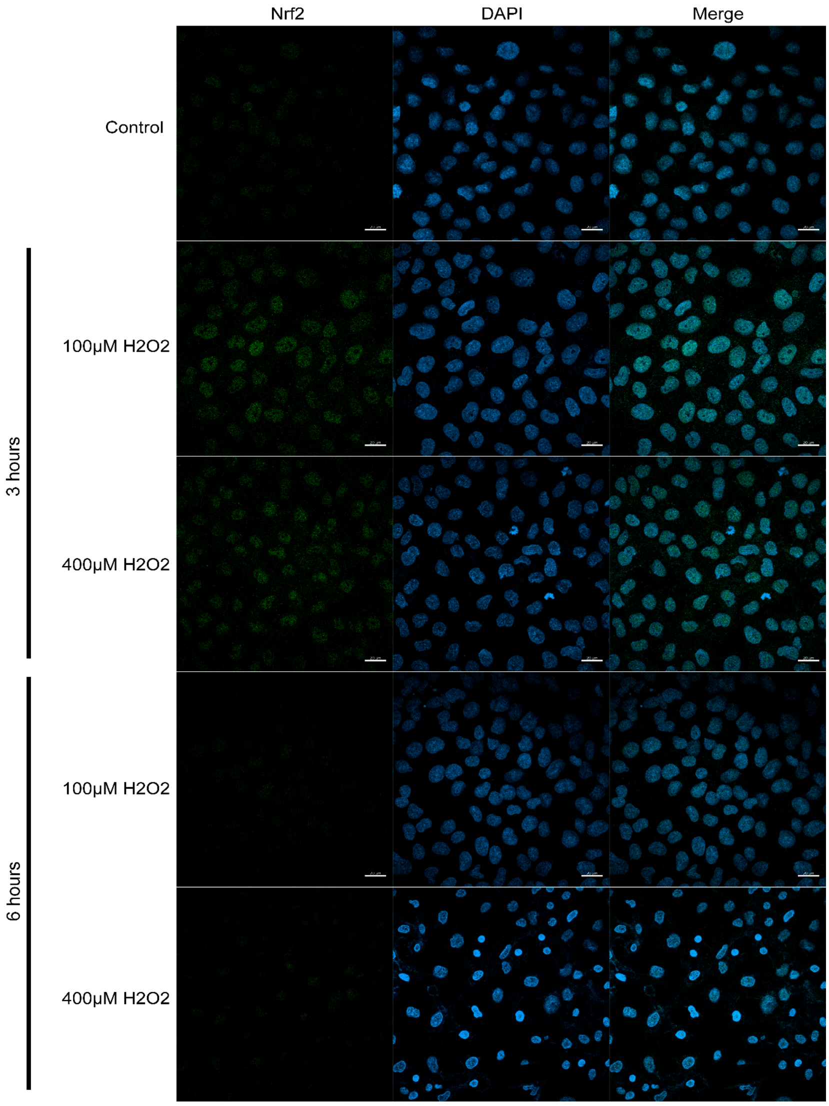

Supplementary #1: Effect of H_2O_2 on Nrf2 pathway in GES-1 cells. This qualitative experiment examined the translocation of Nrf2 to the nucleus. GES-1 cells were exposed to 100 and 400 µM H2O2 for 3 hours and 6 hours, labeled with primary antibodies for Nrf2 (and secondary Alexa Fluor 488 in the green channel), and stained with DAPI (in the blue channel). Note that Nrf2 labeling increased at 3 hours but decreased at 6 hours. The decreased translocation of Nrf2 at 6 h coincides with signs of nucleus damage (pyknotic nuclei) induced by H_2O_2.

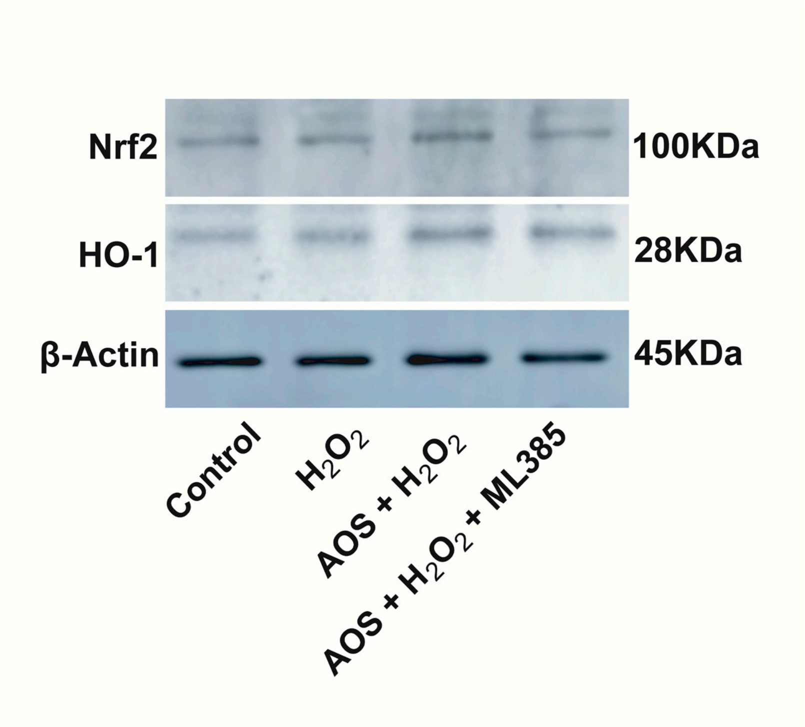

Supplementary Figure 2: Effect of ML385 on Nrf2 pathway via Western blotting. GES-1 cells were treated similarly to those described in Figure 4A and then lysed and processed for Western blotting analysis of Nrf2 and HO-1 proteins.
